# Altering Facial Movements Abolishes Neural Mirroring of Facial Expressions

**DOI:** 10.3758/s13415-021-00956-z

**Published:** 2021-10-12

**Authors:** Kayley Birch-Hurst, Magdalena Rychlowska, Michael B. Lewis, Ross E. Vanderwert

**Affiliations:** 1 School of Psychology, Cardiff University, 70 Park Place, Cardiff, CF10 3AT UK; 2 School of Psychology, Queen’s University Belfast, Belfast, BT7 1NN UK; 3 Cardiff University Centre for Human Developmental Science (CUCHDS), Cardiff University, Cardiff, CF10 3AS UK; 4 Cardiff University Brain Research Imaging Centre (CUBRIC), Cardiff University, Cardiff, CF24 4HQ UK

**Keywords:** Facial mimicry, Facial expression, Mu rhythm, Sensorimotor simulation

## Abstract

People tend to automatically imitate others’ facial expressions of emotion. That reaction, termed “facial mimicry” has been linked to sensorimotor simulation—a process in which the observer’s brain recreates and mirrors the emotional experience of the other person, potentially enabling empathy and deep, motivated processing of social signals. However, the neural mechanisms that underlie sensorimotor simulation remain unclear. This study tests how interfering with facial mimicry by asking participants to hold a pen in their mouth influences the activity of the human mirror neuron system, indexed by the desynchronization of the EEG mu rhythm. This response arises from sensorimotor brain areas during observed and executed movements and has been linked with empathy. We recorded EEG during passive viewing of dynamic facial expressions of anger, fear, and happiness, as well as nonbiological moving objects. We examine mu desynchronization under conditions of free versus altered facial mimicry and show that desynchronization is present when adult participants can freely move but not when their facial movements are inhibited. Our findings highlight the importance of motor activity and facial expression in emotion communication. They also have important implications for behaviors that involve occupying or hiding the lower part of the face.

**Supplementary Information:**

The online version contains supplementary material available at 10.3758/s13415-021-00956-z.

## Introduction

We see facial expressions very frequently. Translating them into meaningful knowledge about other people’s emotions and empathizing with others is essential to our social functioning. How do people process facial expressions to understand others’ mental states? Despite the importance of empathy and emotion recognition in interpersonal and intergroup exchanges (Cikara et al., 2011), the processes underlying that ability are yet to be explained. Identifying such mechanisms is key to understanding typical and atypical development of social cognition.

A growing body of evidence suggests that we can interpret and share other people’s emotions through sensorimotor simulation (Ferrari & Coudé 2018; Wood et al., 2016b). In that process, seeing an emotion expression triggers reactions associated with the production of that expression in the observer’s brain. Such simulation gives the perceiver access to the emotional experience underlying the facial expression, enabling them to infer what the expresser is feeling and empathize with another person’s experience.

Sensorimotor simulation is linked to facial mimicry, or imitation of the facial expressions by the perceiver (Wood et al., 2016a; Wood et al., 2016b). A large body of evidence (Dimberg et al., 2000) shows that viewing facial expressions elicits quick and unconscious facial movements matching the observed expressions. Such reactions have been linked with emotion understanding (Hess & Fischer 2013). They also can be modulated by many factors (Kraaijenvanger et al., 2017 and Seibt et al., 2015 for review), including social or mechanical reasons. For example, people tend to imitate facial expressions of likeable individuals and fellow members of the same group more than the expressions displayed by disliked individuals or members of other groups (Bourgeois & Hess 2008; Likowski et al., 2008; van der Schalk et al., 2011, although see also Hühnel et al., 2014, Peng et al., 2020, and Sachisthal et al., 2016). Moreover, in daily life facial expressions can be hidden by head coverings (Fischer et al., 2011), pacifiers in infants (Rychlowska et al., 2014a), or recently, by face masks (Carbon, 2020; Langbehn et al., 2020), and such perceptual occlusion reduces observers’ facial mimicry (Rychlowska et al., 2014a). However, existing research shows that, even when others’ faces are fully visible, blocking or altering facial mimicry can decrease observers’ ability to detect subtle changes in emotional expressions (Niedenthal et al., 2001), impair accurate categorization of emotion expressions (Oberman et al., 2007; Ponari et al., 2012), and compromise judgments of true or false smiles (Maringer et al., 2011). There is evidence that mimicry-altering manipulations selectively affect recognition of facial expressions that rely on the muscles affected by the manipulation. For example, experimental procedures involving the lower half of the face are more likely to impair recognition of facial expressions of happiness or disgust, as these expressions involve marked activity in the mouth region (Borgomaneri et al., 2020; Oberman et al., 2007; Ponari et al., 2012).

Although the link between facial movements and emotion processing is not always consistent (Bogart & Matsumoto, 2010; Hess & Fischer, 2013; Holland et al., 2020), meta-analyses(Hess & Fischer, 2013) support the association between facial mimicry, emotional experience, and facial expression recognition Coles et al., 2019. Variations in the effects can be due to the existence of multiple pathways to recognizing facial expressions. Beyond sensorimotor simulation, those pathways can include visual information or knowledge about the social context (Adolphs, 2002; de la Rosa et al., 2018). However, existing findings suggest that emotional experience at least partly relies on activation of facial muscles and highlight the need to understand how facial mimicry supports emotion processing. It is thus important to examine the relation between facial movements and the processes that support emotion understanding.

Manipulations of facial mimicry have been shown to influence a wide range of abilities, including perception and categorization of other people’s facial expressions (Lewis & Dunn, 2017; Quettier et al., 2021; Wood et al., 2016a); representations of facial expressions in visual working memory (Sessa et al., 2018), or semantic processing of emotional language (Davis et al., 2015). In addition, interfering with facial movements changes neural responses to emotion expressions. In an experiment using functional magnetic resonance imaging, Hennenlotter et al. (2009) showed that temporary facial paralysis induced by Botox injections changed observers’ reactions to expressions of anger by reducing the activation of amygdala and brain stem areas associated with anger arousal. Studies measuring electrical brain activity with electroencephalography (EEG) suggest that interfering with facial mimicry influences event-related potential responses, such as P1 and N170, which reflect early visual processing (Achaibou et al., 2008; Lomoriello et al., 2021), sustained posterior contralateral negativity associated with visual working memory (Sessa et al., 2018), and N400 linked with semantic processing (Davis et al., 2015, 2017).

Among EEG neural responses, desynchronization of the mu rhythm is particularly relevant to sensorimotor simulation and emotional mirroring. Mu desynchronization arises from sensorimotor brain areas (Hobson & Bishop, 2016) and has been proposed as an indicator of the activity of human mirror neuron system (MNS; Fox et al., 2016; Hobson & Bishop, 2016). The MNS is a network of brain areas containing a special class of neurons responding similarly to perceived, executed, or imagined motor actions (Gallese et al., 2004; Vanderwert et al., 2013). The mu rhythm is defined as electric activity in the 8-13Hz frequency range recorded at central electrodes overlaying the sensorimotor cortex. When at rest, cells in this brain area fire in synchrony leading to higher power in the mu frequency band. However, when an action is performed, observed, or imagined, firing of the cells becomes desynchronized leading to event-related mu desynchronization (Fox et al., 2016).

Mu desynchronization has been observed during the first-hand experience of pain as well as observation of pain in others (Cheng et al., 2008; Yang et al., 2009), during observation and execution of hand gestures (Muthukumaraswamy et al., 2004), and, importantly, facial expressions. For example, Moore et al. (2012) found significant mu desynchronization in adults while they viewed happy and disgusted faces but not when they viewed buildings or visual noise. Subsequent research supported those findings by showing mu desynchronization to emotional expressions in adults (Krivan et al., 2020; Moore & Franz, 2017) and infants (Rayson et al., 2016).

Given that facial mimicry is thought to reflect sensorimotor simulation of perceived expressions (Wood et al., 2016a), it is reasonable to expect links between mimicry and mu desynchronization. However, to our knowledge, only one study has examined that relationship. Specifically, Bernier and colleagues (Bernier et al., 2013) found a positive correlation between mu desynchronization and accuracy of imitation of facial movements. The current study is the first to investigate how altering mimicry affects mu desynchronization to facial expressions. We recorded EEG brain activity of 38 adult participants to videos presenting fearful, happy, and angry expressions as well as nonbiological movement. Participants viewed the videos under conditions of free and altered facial mimicry. In the altered mimicry condition, subjects were asked to hold a pen in their mouth following an established procedure for inhibiting facial movements (Figure 2A; Borgomaneri et al., 2020; Oberman et al., 2007). Consistent with existing findings, we predicted that observation of facial expressions would elicit significant mu desynchronization relative to a baseline period, whereas observation of nonbiological stimuli would not. Crucially, we also hypothesized that interfering with facial mimicry would significantly reduce mu desynchronization to facial expressions but not to nonbiological movement. Finally, we explored whether the potential effects of altering mimicry on mu desynchronization are moderated by participants’ facial recognition abilities measured in a task where participants sorted photographs of happy, sad, fearful, angry, and neutral facial expressions into categories.

## Methods

### Participants

Fifty-four healthy adults (6 males, age *M* = 21.04 years, *SD* = 2.76) completed the task and were compensated with course credit. Our goal was to recruit at least 50 participants, anticipating a 25% rate of data loss due to various elements to the experiment, including suboptimal EEG data. The experiment was approved by the Cardiff University School of Psychology Ethics Committee [EC.17.02.14.4832GRA4]. Sixteen female participants were excluded from the analysis because of technical difficulties (*N* = 2) or because subjects moved to the extent that interfered with the task (*N* = 3), did not provide a minimum of 3 artifact-free trials per condition (*N* = 4), or were statistical outliers (values of mu desynchronization in the central cluster exceeding 3 standard deviations from the *sample* mean in any experimental condition, *N* = 7).^1^ The final sample included 38 participants (6 males, age *M* = 21.21, *SD* = 3.16).

### Materials and Stimuli

#### Emotion Recognition Task

**Fig. 1:**
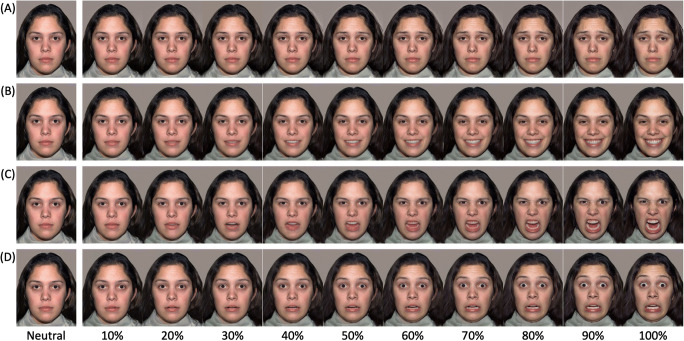
Stimuli used in the emotion recognition task. Participants sorted photographs of sad (**A**), happy (**B**), anger (**C**), and fear (**D**) expressions at increasing 10% intensity levels (NimStim Model 03) into neutral, sad, happy, anger, and fear categories

Stimuli were photographs of two females displaying expressions of happiness, sadness, fear, and anger, plus a neutral face, selected from the NimStim Face Stimulus Set (models 09 and 10; Tottenham et al., 2009). For each model, each emotion expression was morphed with the neutral face to create 10 intensity levels ranging from 10 to 100% (see Gao & Maurer, 2010). This resulted in 44 images for each model (4 expressions x 10 intensities + 4 neutral faces) giving a total of 88 stimuli (see Figure 1). Each image was printed in color (size: 9.5 x 12 cm), stuck onto card, and laminated.

The task was based on of the procedure used by Gao and Maurer (Gao & Maurer, 2010). Briefly, participants were asked to put the stimuli cards into five boxes corresponding to each emotion and marked with a schematic face on the front. The task served to compute scores of participants’ emotion recognition abilities. Scores were based on angry, fearful, and happy faces, as only these are relevant to the EEG task. Analyses using the full stimulus set are reported in Supplementary Materials. To compute scores for each participant, we recorded the number of correct responses for each emotion and then divided by 20 (the total number of possible cards per emotion). Participants were generally consistent in their performance across stimulus types and achieved perfect labeling accuracy between 20% and 30% intensity. To obtain overall emotion recognition scores, we averaged each participants’ accuracy for angry, fearful, and happy expressions into a single score. We then used the grand median value of emotion recognition scores (0.77) to divide participants in two groups: the “High Accuracy” group (*n* = 18) and the “Low Accuracy” group (*n* = 20), see Table 1 for descriptives.

**Table 1:** Mean accuracy scores (proportion of correct responses) for each stimulus type for high- and low-accuracy performers

	Anger	Fear	Happy	Overall
Low accuracy	0.79 (0.05)	0.76 (0.07)	0.68 (0.06)	0.75 (0.02)
High accuracy	0.84 (0.06)	0.90 (0.07)	0.76 (0.06)	0.83 (0.04)

*Note.* Standard deviation in parentheses.

#### EEG Task

**Fig. 2:**
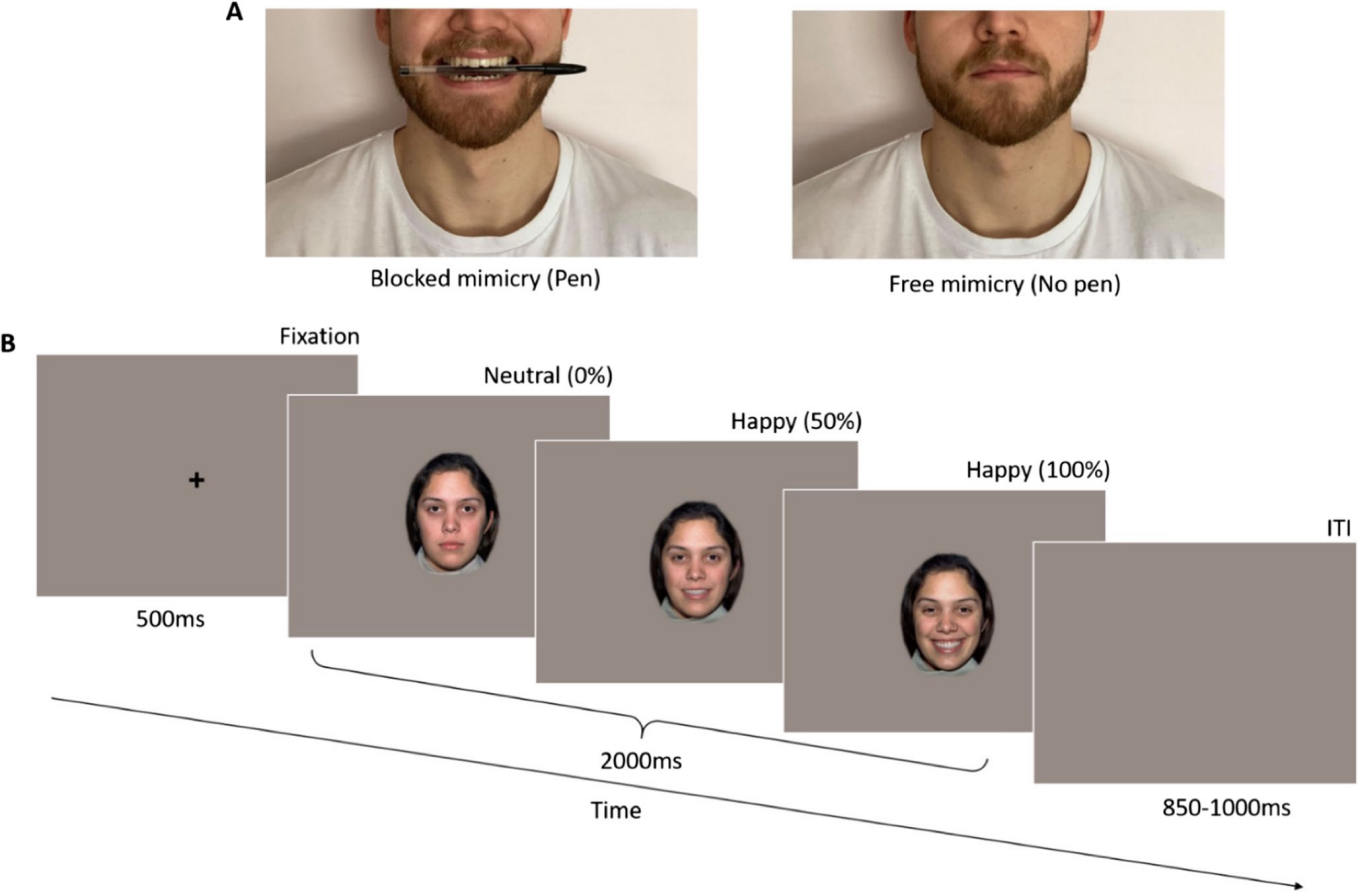
Experimental design. (**A**) For half of the trials, participants held a pen between their teeth to alter their facial mimicry (left) and sat relaxed without the pen (right) for the other half. (**B**) Each trial began with a fixation cross followed by the dynamic video stimulus that played for 2,000 ms, ending with a variable length blank screen intertrial interval (ITI). Videos started with a neutral expression and ended with the full-intensity expression. Control trials displayed animations of nonsocial objects

We recorded participants’ EEG activity during passive viewing of dynamic facial expressions and nonbiological moving objects. Stimuli were 2,000-ms videos of two females from the NimStim set (models 07 and 08; Tottenham et al., 2009) and presented their neutral faces changing to intense (100%) happy, angry, and fearful facial expressions. To create the stimuli, we morphed between each 10% intensity expression images described earlier to show a smooth continuum of 1% changes. We included videos of nonbiological motion as a control condition. These sequences also lasted 2,000 ms and showed animations of 5 nonsocial objects: a ball, rattle, cartoon cat, cartoon duck, and a toy worm (Tobii). Stimuli were presented on a grey background and faces subtended a viewing angle of 15.7° for height and 12.4° for width. Each trial began with a fixation cross appearing for 500 ms, followed by the stimulus lasting 2,000 ms and by an intertrial interval ranging from 850 to 1,000 ms (Figure 2B). Participants saw 20 repetitions of each stimulus, for a total of 160 trials (2 blocks x 80 stimuli, in each block 20 happy, 20 angry, 20 fearful, 20 nonbiological motion^2^).

### Procedure

Participants were tested individually. After reading the information sheet and providing consent, participants completed the emotion recognition task followed by a 5-minute break. They then moved to another room for the capping and EEG task. They sat approximately 65 cm away from a 17” computer screen. E-Prime 2.0 Professional software was used for stimulus presentation. Before the test trials began, we collected 6 minutes of resting EEG data.

After this procedure, participants were randomly assigned to start the experiment either with the pen block or the no-pen block. In the no-pen block, participants received the standard instructions for the EEG procedure. During the pen block, subjects were asked to hold a pen in their mouth. Each participant received a new pen and was instructed to place it in their mouth horizontally and hold it with their teeth without allowing their lips to touch the pen (Borgomaneri et al., 2020; Oberman et al., 2007). Before the beginning of the pen block, the experimenter demonstrated the correct way of holding the pen. The order of blocks was counterbalanced and only one model was shown in each block. Throughout the EEG task, subjects were reminded to sit still and to avoid blinking during the test trials. At random intervals (10 times in each block), participants saw screens with messages asking them to take a break and blink if needed. These screens also asked whether the last stimulus they saw was positive or negative and prompted to answer with a button press. Responses to this question were not recorded as they served to maintain participant engagement with the task and to give subjects the opportunity to take a short break if needed. The two blocks of the EEG task lasted approximately 40 minutes.

### EEG Data Acquisition and Processing

Continuous EEG was recorded with the BrainVision actiCHamp Plus system (Brain Products GmbH, Gilching, Germany) from 32 channels placed according to the International 10-20 System and referenced to the vertex (Cz). ECI Electro-Gel (GVB geliMED GmbH, Germany) was used to improve conductivity. Data were sampled at 500 Hz. All EEG channel impedances were kept below 10 kΩ at the start of data acquisition.

Recorded EEG data were preprocessed using MATLAB. Data were band-pass filtered from 0.5-100 Hz and then re-referenced to the average of all used channels. We extracted epochs from 250 ms before stimulus onset to 2,000 ms after stimulus onset. Epochs containing eye blinks and other movement artifacts (channels exceeding ±100 μV) were identified and excluded from further analysis. With these parameters, we removed an average of 5.42 trials (*SD* = 3.39) in each of the 8 experimental conditions (2: mimicry manipulation x 4: stimulus type). Due to generally poor signal quality across multiple participants, channels FT9, T7, TP9, FT10, T8, and TP10 were excluded from all analyses.

To compute values of event-related mu desynchronization (ERD), EEG signal was band-pass filtered for the adult alpha rhythm (8-13 Hz) and then squared to produce power values (μV^2^) before averaging trials within each stimulus condition. For all clean epochs identified, we computed average power in 250-ms bins over a period of 2,000 ms corresponding to the stimulus presentation and baseline corrected to the last 250 ms of the fixation cross. A positive value indicates event-related mu synchronization whilst a negative score indicates mu desynchronization.

We calculated ERD values for each participant, separately for each experimental condition and averaged across the primary channels of interest with respect to mu desynchronization: the central, central-parietal and parietal electrodes (C3, CP5, P3 and C4, CP6, P4; left and right hemisphere, respectively). To explore alpha desynchronization in the visual cortex and distinguish mu desynchronization from alpha-band activity reactive to visual simulation and attention, we used data from the occipital electrodes O1 and O2. Preliminary analyses indicated no significant effect of hemisphere on mean ERD values in either region, therefore we averaged activity from the right and left electrodes for both the central and occipital electrodes to form a central cluster (C3, CP5, P3, C4, CP6, P4) and occipital cluster (O1, O2). Previous studies examining facial emotion processing using emotion recognition tasks have shown that adult participants typically reach ceiling performance at approximately 50% intensity or above (Gao & Maurer, 2010), therefore, to examine emotion-related effects we made an a priori decision to average mu desynchronization values across the four 250-ms bins corresponding to a change from 50% intensity to peak intensity (1,000-2,000 ms).

To be included in the analysis, participants needed to provide at least three artifact-free trials in each of the eight experimental conditions (2: mimicry manipulation x 4: stimulus type). On average, participants completed 15.76 (*SD* = 3.60) Anger, 15.53 (*SD* = 3.03) Fear, 14.97 (*SD* = 4.03) Happy, and 13.08 (*SD* = 4.00) nonbiological artifact-free trials in the No Pen conditions and 15.18 (*SD* = 4.05) Anger, 14.58 (*SD* = 3.93) Fear, 14.79 (*SD* = 4.49) Happy, and 12.71 (*SD* = 4.96) nonbiological artifact-free trials in the Pen conditions. There were no significant differences between the Pen and the No Pen blocks in number of completed trials for any emotion.

### Statistical Analysis

We first sought to establish mu desynchronization to facial expressions. For this, we used one-sample*t*-tests to compare ERD in the central cluster against a baseline of zero, separately for each of the eight experimental conditions. In addition, we examined the effects of mimicry manipulation (pen, no pen) and stimulus type (anger, fear, happy, nonbiological) on ERD in the central electrode cluster using a 2 x 4 within-subjects ANOVA. To distinguish mu desynchronization from visual processing, we used an identical analysis to examine ERD in the occipital cluster as a function of stimulus type and mimicry manipulation. Finally, we used a within-subjects ANOVA to examine mu desynchronization in central clusters as a function of emotion recognition accuracy (low, high), mimicry manipulation (pen, no pen), and time (0-500 ms, 500-1,000 ms, 1,000-1,500 ms, 1,500-2,000 ms). All post-hoc tests were Bonferroni corrected. We used the Greenhouse-Geisser correction when assumption of sphericity was violated.

## Results

### EEG Task

#### Mu Desynchronization to Facial Expressions

**Fig. 3:**
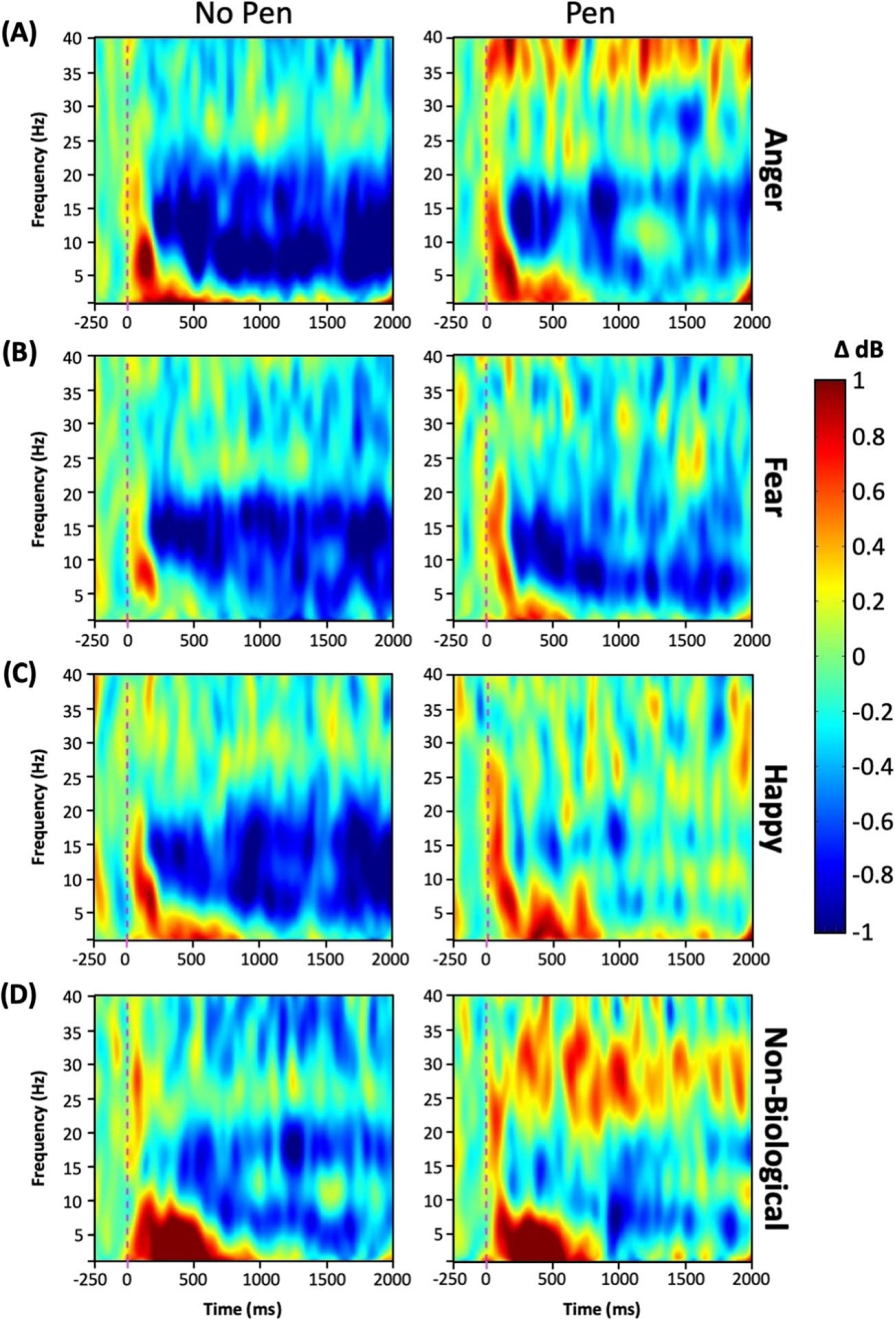
Time-frequency plot in the central cluster for **(A)** anger, **(B)** fear, **(C)** happy, and **(D)** nonbiological stimuli for (left) no pen and (right) pen conditions over the duration of the stimulus presentation. Cool colors reflect event-related desynchronization and warm colors synchronization with respect to baseline

Table 2 (left panel) displays mean ERD values per stimulus type in the free mimicry block. One-sample*t*-tests comparing ERD values in each condition against a baseline of zero desynchronization revealed significant mu desynchronization in reaction to anger, fear, and happiness but not to nonbiological movement. Figure 3 displays time-frequency plots for the central cluster for each stimulus type.

**Table 2:** Mu desynchronization in the free mimicry (no pen) and altered mimicry (pen) conditions

Stimulus type	No pen		*t*	*Sig.*	Pen		*t*	*Sig.*
	*M*	*SD*			*M*	*SD*		
Anger	−1.01	1.82	3.41	<0.001^***^	−0.06	1.14	0.34	0.367
Fear	−0.77	1.50	3.19	0.001^**^	−0.52	1.77	1.82	0.038^*^
Happy	−0.48	1.53	1.94	0.030^*^	0.07	1.52	0.29	0.387
Nonbiological	−0.03	1.97	0.09	0.466	0.02	1.43	0.10	0.462

*Note:*One-tailed*t*-tests compared with zero desynchronization with *df* = 37.

*p < 0.05; **p < 0.01; ***p < 0.001.

#### Effects of Altering Facial Mimicry on Mu Desynchronization

##### Central Cluster

**Fig. 4:**
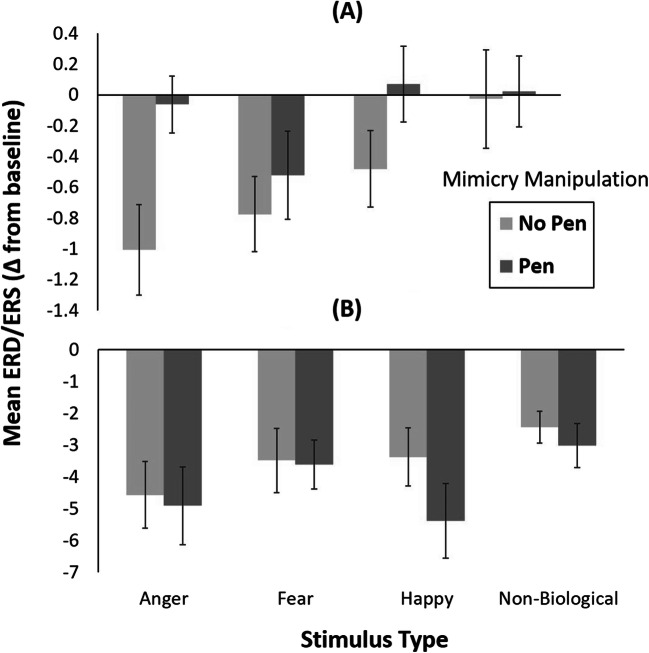
Mean mu desynchronization values for each stimulus type in the pen and no pen conditions for the **(A)** central cluster (C3, CP5, P3, C4, CP6, and P4) and the **(B)** occipital cluster (O1 and O2). Error bars represent ±1 standard error

A repeated measures ANOVA with mimicry manipulation (pen, no pen) and stimulus type (anger, fear, happy, nonbiological) as within-subject variables revealed a significant main effect of the mimicry manipulation, *F*(1, 37) = 5.27, *p* = 0.03, η_p_^2^ = 0.12, with significantly greater mu desynchronization in the no pen condition (*M* = −0.57, *SD* = 1.16) than in the pen condition (*M* = −0.12, *SD* = 0.82; Figure 4A). The main effect of stimulus type was also significant, *F*(3,111) = 3.50, *p* = 0.02, η_p_^2^ = 0.09. Post-hoc pairwise comparisons revealed that mu desynchronization was significantly greater when observing fearful faces (*M* = −0.65, *SD* = 1.25) compared with nonbiological movement (*M* = −0.003, *SD* = 1.31; *t*(37) = 2.88, *p* = 0.02) in the central cluster. All remaining contrasts were not significant (angry vs. fearful: *t*(37) = 0.52, *p* = 1.0; angry vs. happy: *t*(37) = 1.69, *p* = 0.30; angry vs. nonbiological: *t*(37) = 1.96, *p* = 0.17; fear vs. happy: *t*(37) = 2.13, *p* = 0.12; happy vs. nonbiological: *t*(37) = 0.91, *p* = 1.0). There was no significant interaction between mimicry manipulation and stimulus type, *F*(3,111) = 1.28, *p* = 0.28, η_p_^2^ = 0.03.

Table 2 (right panel) displays mean ERD values per stimulus type in the altered-mimicry pen condition. One-sample t-tests against the baseline of 0 revealed that significant mu desynchronization occurred only to fearful expressions but not to other types of stimuli.

##### Occipital Cluster

Attention-related alpha desynchronization in the occipital electrodes O1 and O2 was also analyzed as a function of mimicry manipulation and stimulus type. The repeated-measures ANOVA revealed a significant main effect of stimulus type, *F*(3, 111) = 3.88, *p* = 0.02, ε = 0.80, η_p_^2^ = 0.10. Post-hoc pairwise comparisons revealed that ERD was significantly greater to angry faces (*M* = −4.75, *SD* = 5.86) than to non-biological movement (*M* = −2.73, *SD* = 3.30; *t*(37) = 3.33, *p* = 0.006) (Figure 4B). All remaining comparisons were not significant (angry vs. fearful: *t*(37) = 1.90, *p* = 0.19; angry vs. happy: *t*(37) = 0.45, *p* = 1.0; fear vs. happy: *t*(37) = 1.52, *p* = 0.41; fear vs. nonbiological: *t*(37) = 1.63, *p* = 0.34; happy vs. non-biological: *t*(37) = 2.23, *p* = 0.10). The main effect of mimicry manipulation and the interaction between mimicry manipulation and stimulus type were not significant, *F*(1, 37) = 1.67, *p* = 0.21, η_p_^2^ = 0.04 and *F*(3, 111) = 0.47, *p* = 0.63, ε = 0.68, η_p_^2^ = 0.01, respectively.

### Emotion Recognition Task and Mu Desynchronization

**Fig. 5:**
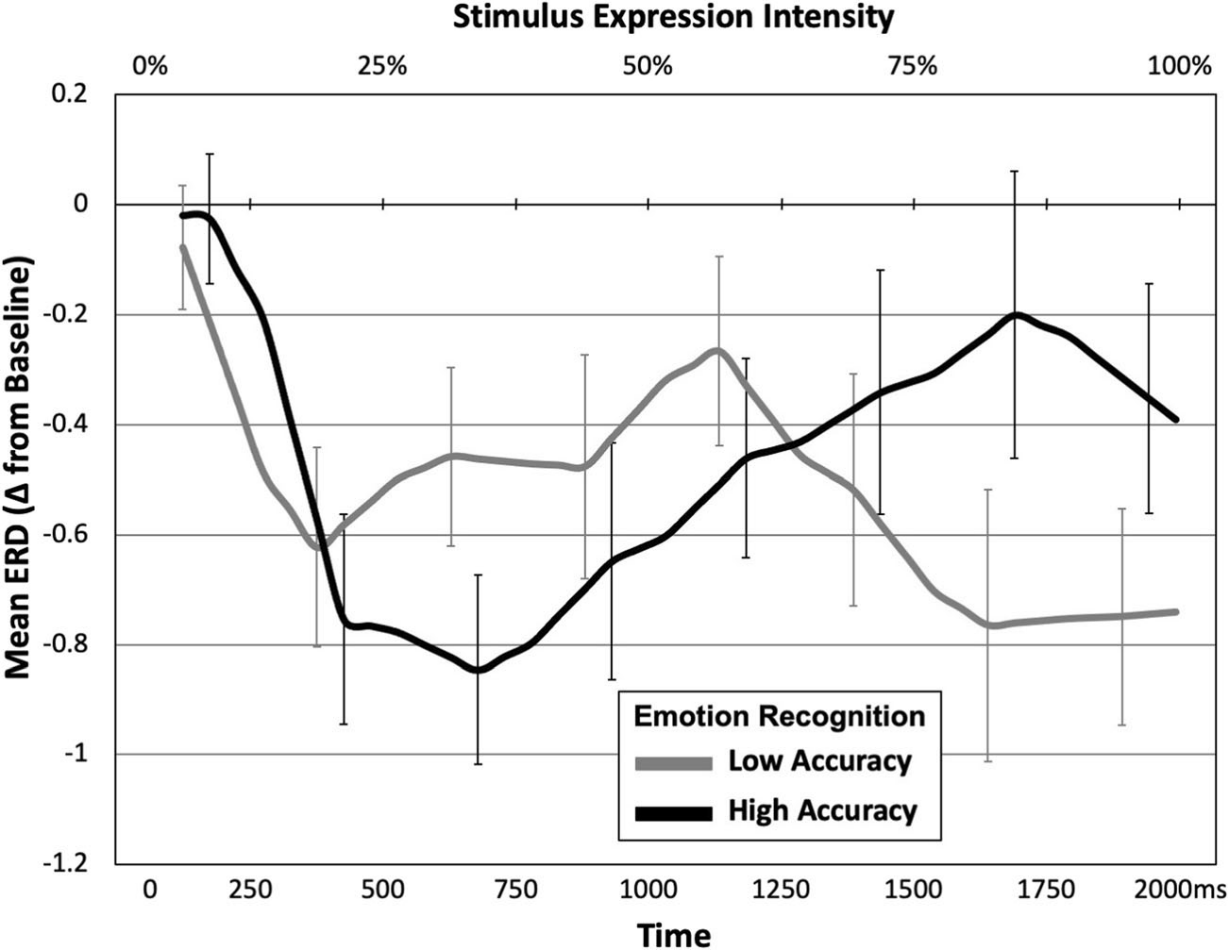
Temporal changes in mu desynchronization to emotion faces for high- and low- accuracy performers on the emotion recognition task. Error bars represent ±1 standard error

The final analysis explored whether the effects of stimulus type and mimicry manipulation on mu desynchronization were moderated by participants’ emotion recognition abilities. Detailed analyses of participants’ emotion recognition performance can be found in the supplementary materials. To examine relations between performance in emotion recognition (high- vs. low-accuracy performers) and temporal changes in mu rhythm in the central clusters, we computed the averaged activity to the face stimuli in these clusters over pairs of 250-ms bins (i.e., 0–500 ms, 500–1,000 ms, 1,000–1,500 ms, 1,500–2,000 ms). We then conducted a repeated measures ANOVA with emotion recognition accuracy (high, low) as a between-subjects variable and mimicry manipulation (pen, no pen) and time (0-500 ms, 500-1,000 ms, 1,000-1,500 ms, 1,500-2,000 ms) as within-subject variables. This analysis revealed a significant main effect of mimicry manipulation, *F*(1, 36) = 7.44, *p* = 0.01, η_p_^2^ = 0.17 demonstrating overall greater mu desynchronization in no pen (*M* = −0.71, *SD* = 1.03) relative to the pen (*M* = −0.24, *SD* = 0.70) condition. The analysis also revealed a significant interaction between accuracy group and time *F*(3, 108) = 4.75, *p* = 0.008, ε = 0.783, η_p_^2^ = 0.17. Post-hoc pairwise comparisons revealed that in the High-Accuracy group mu desynchronization was greatest for 500-1000ms (*M* = −0.75, *SD* = 0.79) compared with 1,000-1,500 ms (*M* = −0.40, *SD* = 0.80, *t*(36) = 3.61, *p* = 0.003) and 1,500-2,000 ms (*M* = −0.28, *SD* = 0.97, *t*(36) = 3.25, *p* = 0.008), but not for 0-500 ms (*M* = −0.39, *SD* = 0.61, *t*(36) = 2.21, *p* = 0.10). Conversely, in the Low-Accuracy group, ERD was greatest in the 1,500-2,000 ms (*M* = −0.76, *SD* = 0.97) compared with 0-500 ms (*M* = −0.35, *SD* = 0.61, *t*(36) = 2.59, *p* = 0.04) and 1,000-1,500 ms (*M* = −0.39, *SD* = 0.81, *t*(36) = 2.82, *p* = 0.02), but not for 500-1,000 ms (*M* = −0.47, *SD* = 0.79, *t*(36) = 2.10, *p* = 0.13). The main effects of time and emotion recognition accuracy were not significant, *F*(3, 108) = 2.26, *p* = 0.10, ε = 0.78, η_p_^2^ = 0.06 and *F*(1, 36) = 0.03, *p* = 0.87, η_p_^2^ = 0.001, respectively, nor were the interactions between mimicry manipulation and emotion recognition accuracy (*F*(1, 36) = 0.64, *p* = 0.43, η_p_^2^ = 0.02), mimicry manipulation and time (*F*(3, 108) = 2.46, *p* = 0.08, ε = 0.79, η_p_^2^ = 0.06), or the three-way interaction between mimicry manipulation, time, and emotion recognition accuracy (*F*(3, 108) = 0.79, *p* = 0.47, ε = 0.79, η_p_^2^ = 0.02). Figure 5 displays changes in mu desynchronization as a function of time and stimulus expression intensity for each group, across both mimicry conditions.

## Discussion

We investigated whether interfering with facial mimicry affects mu desynchronization, an EEG response indexing neural mirroring and emotion resonance, in reaction to angry, happy, and fearful facial expressions. When participants could freely move their face, observation of emotion expressions, but not nonbiological moving stimuli, elicited significant mu desynchronization relative to a baseline. Importantly, inhibiting the movements of the lower half of the face abolished mu desynchronization to happy and angry expressions. Moreover, the time course of mu desynchronization depended on participants’ emotion recognition abilities such that high-accuracy performers showed peak desynchronization earlier than low-accuracy performers.

In line with previous research (Krivan et al., 2020; Moore et al., 2012; Moore & Franz, 2017; Rayson et al., 2016), these findings replicate evidence that viewing emotional faces elicits an activation of sensorimotor brain areas reflected by mu desynchronization. We observed such reaction when participants watched facial expressions but not non-biological objects, suggesting that mu desynchronization is specific to facial movements rather than moving stimuli in general (Aleksandrov & Tugin, 2012; Hobson & Bishop 2016).

Our findings reveal that disrupting facial movements (Borgomaneri et al., 2020; Oberman et al., 2007) abolishes mu desynchronization to emotional faces. Importantly, that effect was observed in central, but not in occipital, regions, indicating sensorimotor processes rather than alpha activity arising in occipital brain regions and associated with attentional engagement (Klimesch, 1999).

We found greater mu desynchronization when participants could freely move their face than in the pen condition interfering with facial movements. That effect did not interact with stimulus type, suggesting that altering mimicry reduced mu desynchronization to all facial expressions. Additional analyses revealed that the pen manipulation abolished mu desynchronization to happy and angry expressions, but not the fearful facial expression, which seemed to elicit mu desynchronization even under conditions of altered mimicry. That finding may seem to contradict the results of a previous study (Ponari et al., 2012), in which recognition of fearful expressions was impaired by restricting facial movements. However, it is worth remembering that facial expressions of fear are predominantly recognized using information from the eye region (Smith et al., 2005). Thus, it is possible that participants were simulating the fearful expression without using the lower part of their face, which was occupied by the pen manipulation. Such an interpretation is supported by two studies (Borgomaneri et al., 2020; Oberman et al., 2007), in which altering facial mimicry with a similar procedure impaired recognition of happy, but not fearful expressions. In addition, facial expressions of anger used in the present study involved marked activity in the lower half of the face, (see Figure 1) which, for our participants, was prevented by the pen manipulation.

To our knowledge, this is the first evidence that interfering with facial movements decreases mu desynchronization. Our study extends previous correlational findings (Bernier et al., 2013) and provides important insights into the mechanisms underlying facial expression recognition. According to simulationist models (Wood et al., 2016b), motor and somatosensory systems contribute to the experienced meaning of facial expressions, and the somatosensory cortex is a central structure implicated in emotion recognition (Adolphs, 2002). Moreover, executing motor commands prepares sensory brain cortices to incoming sensory feedback through efference copies (Nelson, 1996), potentially altering somatic sensations and the simulated experience of how a perceived facial expression *feels*. Consistent with the claim that altering facial movements disrupts embodied simulation, mu desynchronization covaries with the activity of several brain regions associated with neural mirroring, including the somatosensory cortex (Vanderwert et al., 2013).

One possible explanation of the lack of mu desynchronization in the pen condition could be that holding the pen in the mouth activated the motor system resulting in either interfering with the mirror neuron system (e.g., Cannon & Woodward, 2008; Gredebäck & Falck-Ytter, 2015; Hommelsen et al., 2017) or by abolishing the mu rhythm before the observation of facial expressions began. This is unlikely to be the case as desynchronization of the mu rhythm due to motor activity induced by the pen condition should have been present in all conditions, including fear faces and the non-biological stimuli. Although none of these effects occurred, we calculated an estimate of mu rhythm power during the baseline period in each condition to exclude the possibility that the pen manipulation altered motor responses prior to the stimuli presentation. The analyses, reported in the supplementary materials, showed no differences in the mu rhythm power between the two pen conditions, suggesting that holding the pen between the teeth did not differentially activate the motor system. Thus, the abolishment of mu desynchronization during the pen block was not due to greater motor activity, but more likely to alterations of facial mimicry in reaction to observed facial expressions.

The present findings dovetail with a large number of studies linking facial mimicry with various stages of emotion recognition, such as early EEG responses reflecting visual processing (Lomoriello et al., 2021), perceptual discrimination of facial expressions (Wood et al., 2016b), and maintaining representations of facial expressions in working memory (Sessa et al., 2018). We also show that the time course of mu desynchronization to facial expressions differs depending on emotion recognition abilities. High-accuracy participants showed peak mu desynchronization earlier than low-accuracy participants, suggesting that people with better emotion recognition abilities mirror perceived expressions earlier than those with poorer recognition abilities. The second group also showed more prolonged mu desynchronization, possibly reflecting the need to mirror over a longer time period as the expression becomes more intense.

We show that altering facial mimicry disrupts neural mirroring of facial expressions indexed by mu desynchronization. Our findings also reveal that individual differences in emotion recognition predict the time course of neural mirroring processes, extending previous findings on individual differences and mimicry (Bernier et al., 2013; Lomoriello et al., 2021; Sessa et al., 2018). The present results highlight the need for future research on mu rhythm, including neutral faces as a control condition and using different types of mimicry-altering manipulations (e.g., Niedenthal et al., 2001; Oberman et al., 2007; Rychlowska et al., 2014b). Future studies should also investigate the extent to which mu desynchronization covaries with facial mimicry and recognition of emotion expressions involving different regions of the face (Ponari et al., 2012). Sensorimotor simulation is not always necessary for facial expression recognition (Bogart & Matsumoto 2010; de la Rosa et al., 2018), but it is proposed that that route is especially important when the observed expression is both socially relevant and challenging to classify (Hess & Fischer (2013); for example, during early developmental periods (Rychlowska & Vanderwert, 2020). The present findings provide new insights into the role of bodily movements in facial expression recognition and emotional experience. They also suggest that interfering with facial activity may be detrimental for social interaction and emotional exchange. This has important implications for prolonged inhibition of facial mimicry, such as in the case of facial paralysis (De Stefani et al., 2019), pacifier use in infants (Rychlowska & Vanderwert, 2020), or when facial expressions are obscured from view, for example by a face mask (Langbehn et al., 2021).

## Supplementary Information

ESM 1 (DOCX 1005 kb)

## Footnotes

1 Analyses were reconducted including the outlying participants and the results were consistent with the main findings and conclusions reported.

2 Nonbiological motion stimuli were randomly drawn from the pool of the 5 objects described.
