# Supplementary material for: Altering Facial Movements Abolishes Neural Mirroring of Facial Expressions
Source: Cogn Affect Behav Neurosci. 2021 Oct 12;22(2):316–27. doi: 10.3758/s13415-021-00956-z (PMC8983526; doi:10.3758/s13415-021-00956-z)

ALTERING FACIAL MOVEMENTS ABOLISHES MU RHYTHM

**Altering Facial Movements Abolishes Neural Mirroring of Facial Expressions**

**Supplementary Materials**

Kayley Birch-Hurst,^1^ Magdalena Rychlowska,^2^ Michael B. Lewis,^1^ and Ross E. Vanderwert^1,3,4^

^1^ School of Psychology, Cardiff University, Cardiff, UK

^2^ School of Psychology, Queen’s University Belfast, UK

^3^ Cardiff University Centre for Human Developmental Science (CUCHDS), Cardiff University, UK

^4^ Cardiff University Brain Research Imaging Centre (CUBRIC), Cardiff University, UK

**Author Note**

This research was supported by a BA/Leverhulme Small Research Grant SG161800 (to M. Rychlowska and R. Vanderwert).

Corresponding Author

Ross E. Vanderwert

School of Psychology, Cardiff University

70 Park Place, Cardiff CF10 3AT

United Kingdom

Email: VanderwertR@cardiff.ac.uk

Phone: +44 (0)29 2068 8826

**Contents**

1. **Analysis of Emotion Recognition Performance**
2. **Analysis of Mu Rhythm Power During the Baseline Period**
3. **Emotion Recognition Task and Mu Desynchronization for Each Stimulus Type**
4. **Analysis of Emotion Recognition Performance**

**Figure S1.** Mean recognition accuracy scores at each intensity level for each emotional expression, averaged across the two models seen by each participant.

**Supplementary Figure S1**

*
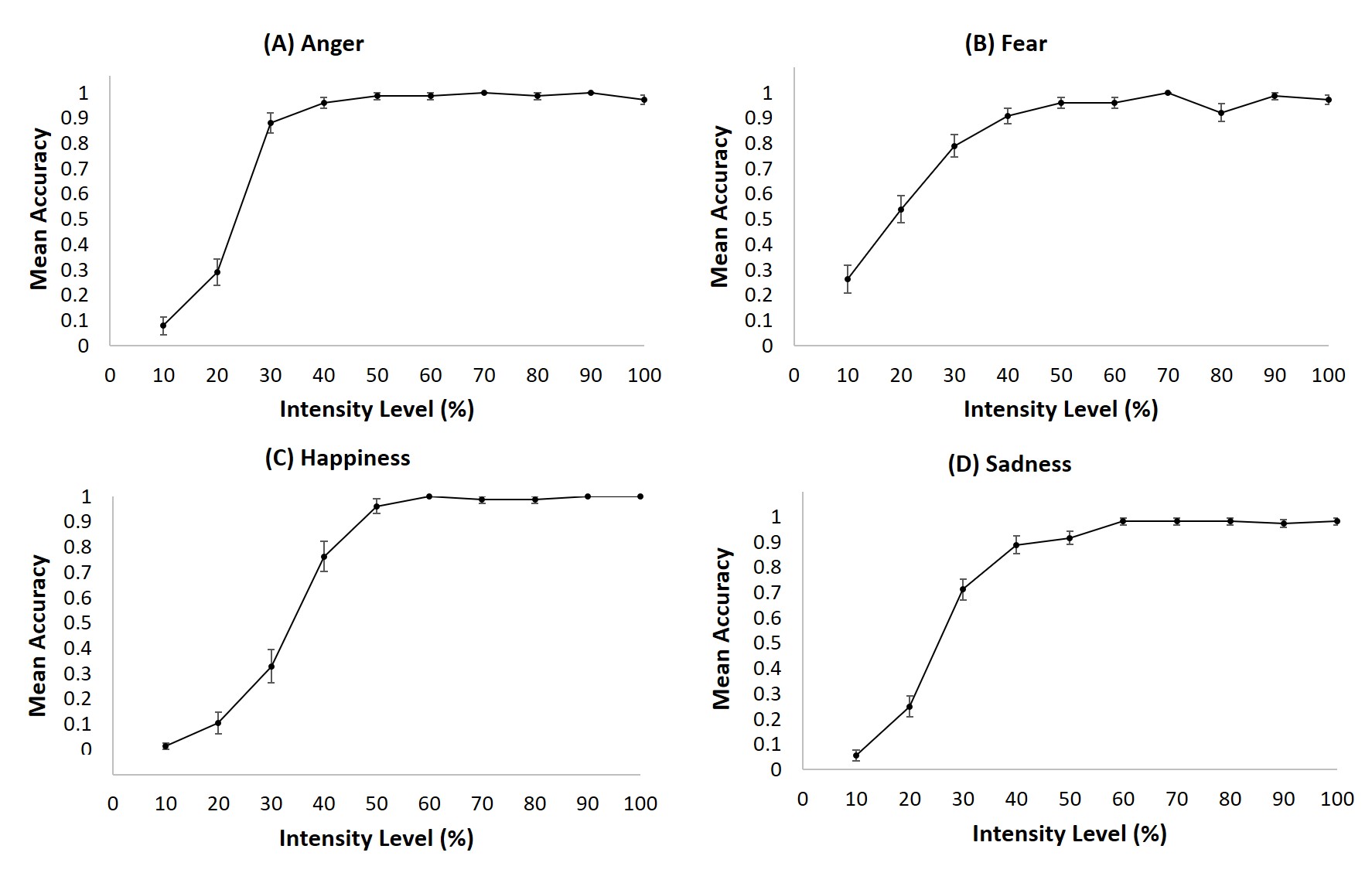
Mean Accuracy Scores for Anger, Fear, Happiness and Sadness as a Function of Intensity*

*Note.* Error bars show ±1 standard error.

Supplementary Table S1 shows the mean number of faces identified as expressive (non-neutral) across all intensity levels for anger, fear, happy and sad expressions. Responses reported in the table include both correct identifications and misidentifications.

Table S2 shows mean levels of intensity necessary to detect each of the four expressions, or minimal intensity levels at which participants were able to correctly label expressive faces as different from neutral. This was defined for each participant as the level at which they no longer made neutral responses.

Table S3 summarises mean misidentification rates. These rates were calculated by dividing the frequency of non-neutral misidentifications by the total number of non-neutral responses for each participant and for each emotion expression.

Misidentification rates above the level of intensity to detect expression in the face were calculated by dividing the frequency of misidentifications by the total number of non-neutral responses for each participant for each emotional expression.

**Supplementary Table S1**

*Mean Number of Photographs (out of 20) Labelled as Non-Neutral for Each Facial Expression*

| **Anger** | **Fear** | **Happiness** | **Sadness** |
| --- | --- | --- | --- |
| 17.5 (87.5) | 17.4 (87) | 14.8 (74) | 16.1 (80.5) |

*Note.* Scores are out of 20 (percentages are in parentheses).

**Supplementary Table S2**

*Average Minimum Intensity Levels Necessary to Detect Each Facial Expression*

| **Anger** | **Fear** | **Happiness** | **Sadness** |
| --- | --- | --- | --- |
| 27.9 (7) | 30.5 (12.5) | 43.4 (10.7) | 35 (8) |

*Note.* Standard deviations are in parentheses.

**Supplementary Table S3**

*Mean Misidentification Rates for Facial Expressions of Anger, Fear and Sadness*

| **Facial expression**  **Misidentified as:** | **Anger** | **Fear** | **Sadness** |
| --- | --- | --- | --- |
| **Anger** | - | 1.9 | 2.9 |
| **Fear** | 3.4 | - | 0.2 |
| **Sadness** | 0.8 | 2.1 | - |
| **Happiness** | 0 | 0 | 0 |
| **Total** | 4.2 | 3.9 | 2.3 |

*Note.* Values are percentages.

1. **Analysis of Mu Rhythm Power During the Baseline Period**

During the normal procedure of averaging neural activity across trials, the average power in the 250ms baseline period would be subtracted from the entire segment (-250ms to 2000ms post stimulus onset); thus calculating the mean power in the averaged baseline period would result in a value of zero µV^2^. To overcome this issue, we followed a modified procedure from that outlined in the manuscript. First the EEG was band-pass filtered for the adult alpha rhythm (8-13Hz). Next, the EEG was segmented from -250ms prior to stimulus onset and 2000ms post stimulus. Each trial was then baseline corrected for the average amplitude of the 250ms pre-stimulus period (Note that at this stage, the EEG signal still has positive and negative deflections). This step ensured that all trials shared a standardized zero-amplitude. The signal in each trial was then squared to produce all positive values to avoid cancelling the non-phase-locked nature of the oscillations with respect to stimulus onset in the averaging process. Finally, all artifact-free trials are averaged together within each stimulus type for each block and the mean power over the baseline period was calculated.

Pairwise t-tests comparing each trial type between blocks of Pen and No Pen were computed for baseline power in each emotion condition. Supplementary Table S4 presents the means, standard deviations, and results of these analyses. There were no significant differences between Pen and No Pen blocks for any of the trial types in either the mu rhythm (Central cluster) or in the alpha rhythm (Occipital cluster).

**Supplementary Table S4**

*Results of Pairwise Comparisons of Baseline Power Between Pen and No Pen Blocks*

|  | Condition | |  |  |
| --- | --- | --- | --- | --- |
|  | No Pen | Pen | *t*(37) | *p* |
| Central |  |  |  |  |
| Anger | 4.35 (3.58) | 4.65 (3.54) | -0.993 | 0.327 |
| Fear | 4.62 (3.95) | 4.35 (2.72) | 0.662 | 0.512 |
| Happy | 4.11 (3.22) | 3.99 (2.53) | 0.362 | 0.719 |
| Non-biological | 4.07 (2.87) | 4.09 (2.98) | -0.082 | 0.935 |
| Occipital |  |  |  |  |
| Anger | 11.68 (12.63) | 11.50 (12.12) | 0.229 | 0.82 |
| Fear | 11.25 (9.21) | 11.12 (8.99) | 0.104 | 0.917 |
| Happy | 10.47 (9.11) | 10.12 (7.08) | 0.412 | 0.682 |
| Non-biological | 9.91 (7.15) | 9.65 (7.01) | 0.488 | 0.628 |
| *Note.* Mean power (µV^2^) are presented with standard deviations in parentheses. *N* = 38. | | | | |

1. **Emotion Recognition Task and Mu Desynchronization for Each Stimulus Type**

Figures S2-S5. Temporal changes in mu desynchronization to (S2) Angry, (S3) Fearful, and (S4) Happy faces and (S5) Non-Biological stimuli for high- and low-accuracy performers on the emotion recognition task. Notes: Accuracy groups were based off overall performance across emotion faces and not specific to the emotion of the displayed neural responses. Non-Biological stimuli were not included in the individual differences analyses reported in the manuscript and do not have a *change in intensity* over the 2000ms window. Please interpret these plots with caution, as the results of the statistical analyses did not warrant examination of the stimulus types independently.

**Supplementary Figure S2**

*Temporal Changes in Mu Desynchronization to Angry Faces for High- and Low- Accuracy Performers on the Emotion Recognition Task. Error Bars Represent ±1 Standard Error.*

**
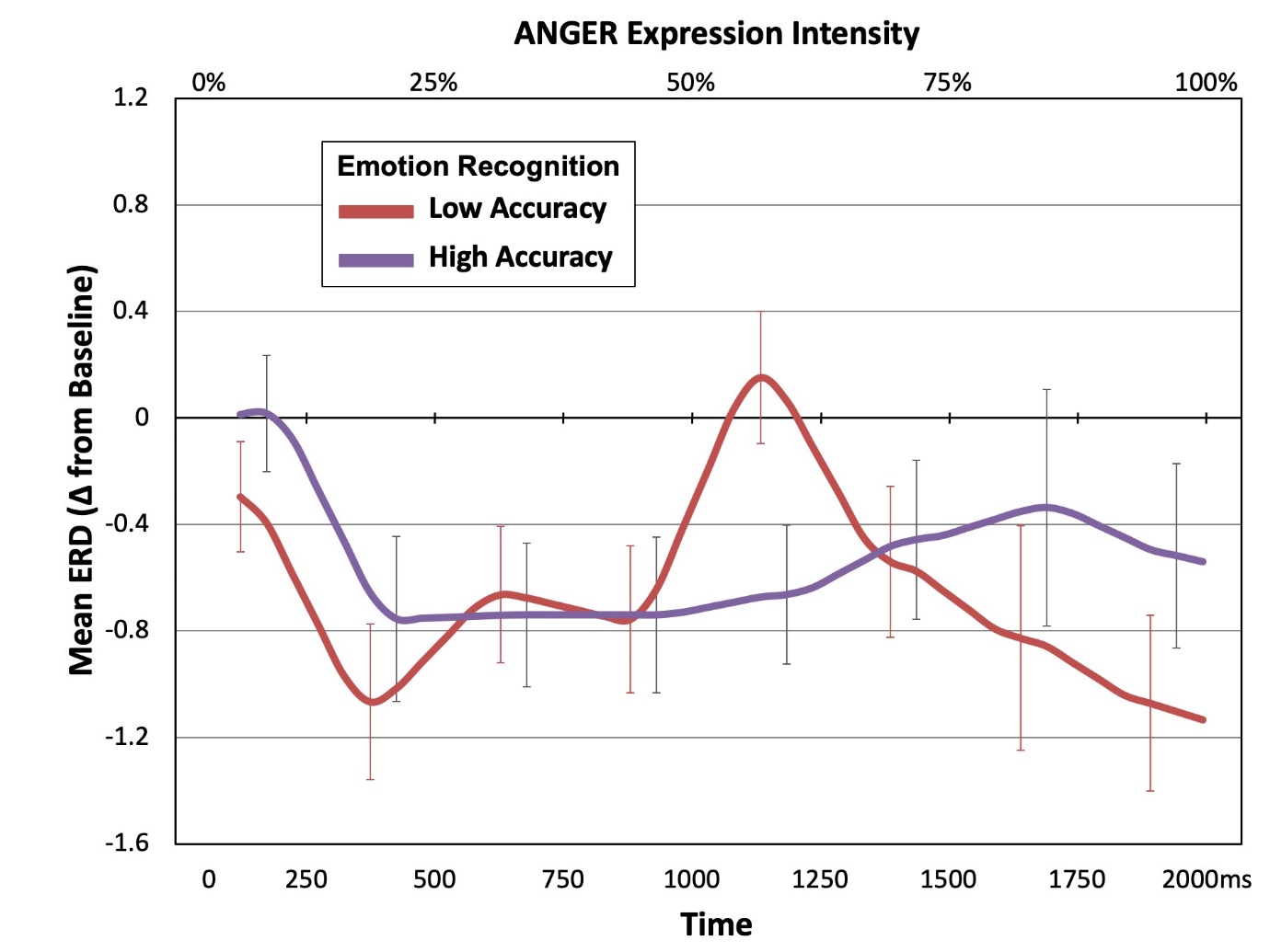
**

**Supplementary Figure S3**

*Temporal Changes in Mu Desynchronization to Fearful Faces for High- and Low- Accuracy Performers on the Emotion Recognition Task. Error Bars Represent ±1 Standard Error.*


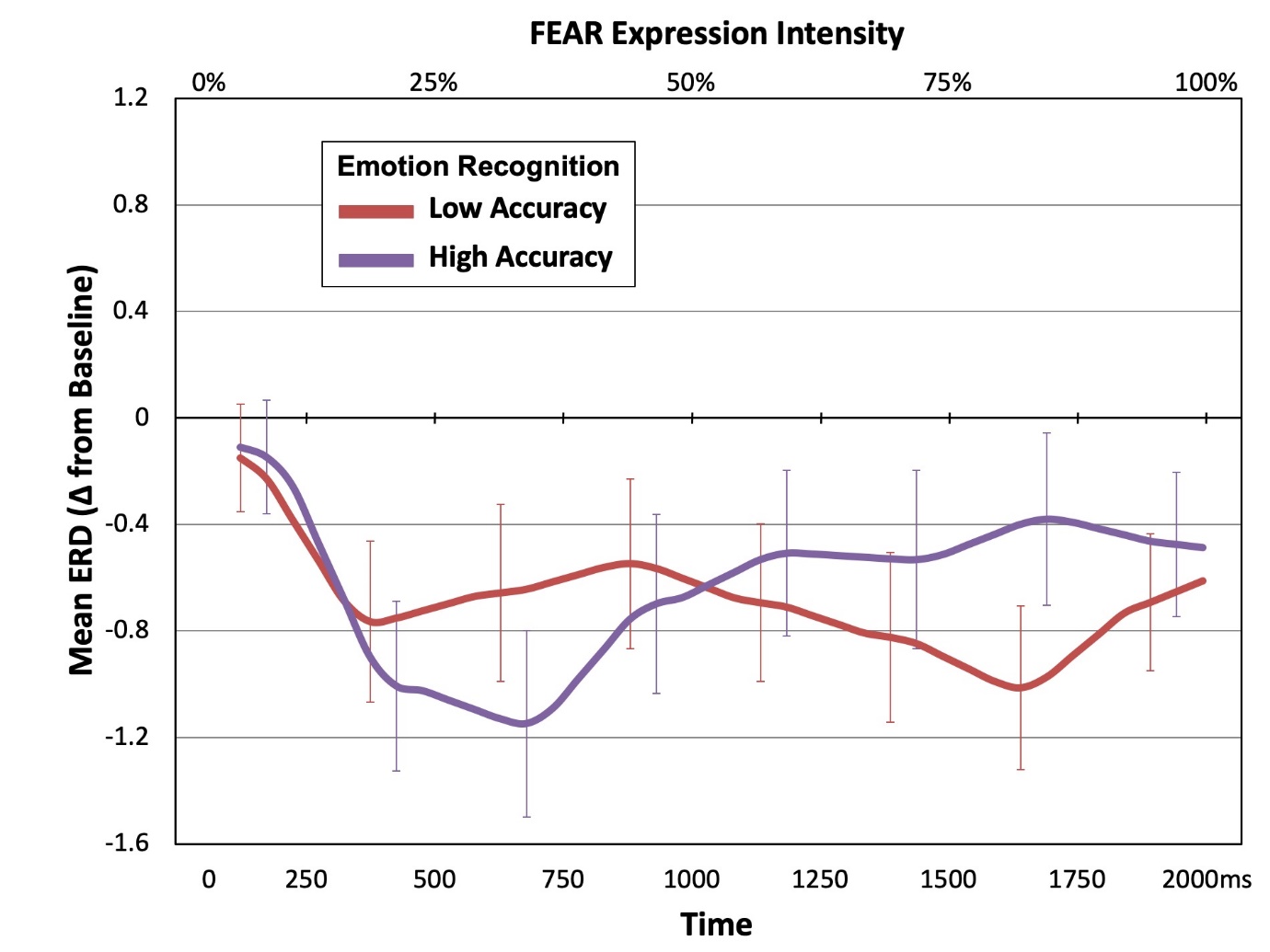


**Supplementary Figure S4**

*Temporal Changes in Mu Desynchronization to Happy Faces for High- and Low- Accuracy Performers on the Emotion Recognition Task. Error Bars Represent ±1 Standard Error.*


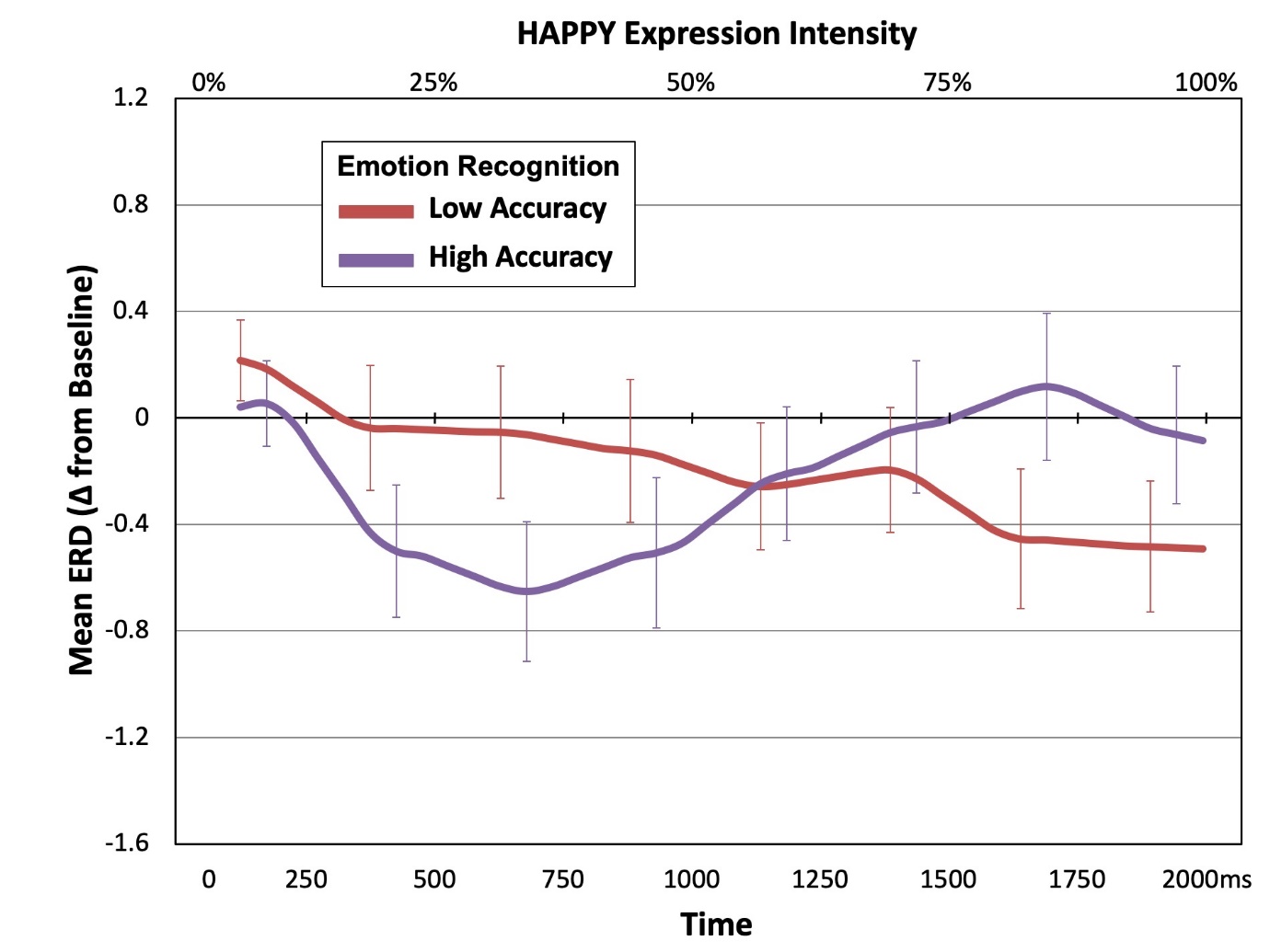


**Supplementary Figure S5**

*Temporal Changes in Mu Desynchronization to Non-Biological Stimuli for High- and Low- Accuracy Performers on the Emotion Recognition Task. Error Bars Represent ±1 Standard Error.*


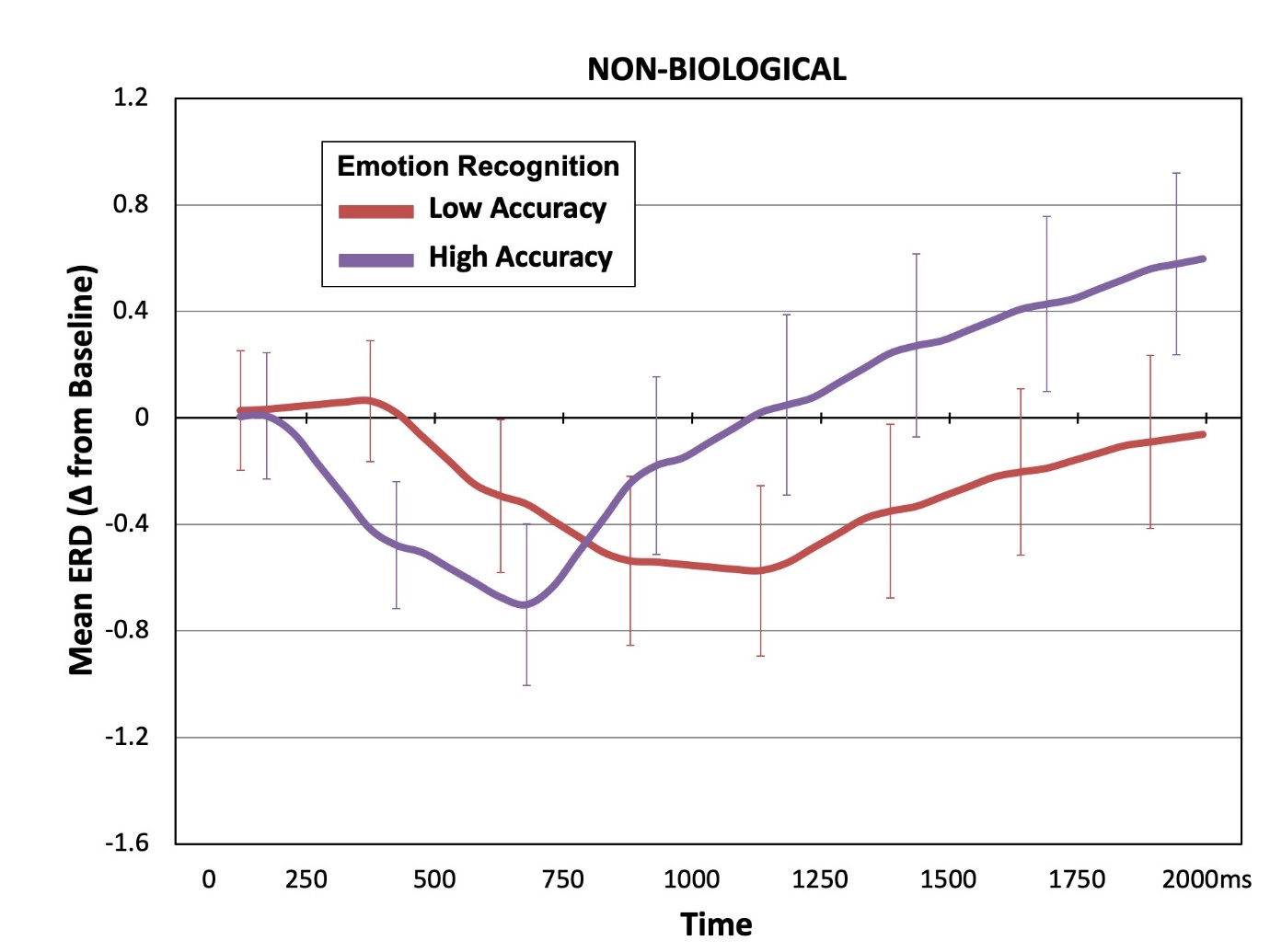


**Supplementary Figure S6**

*Scalp distribution of the change in mu rhythm power for each emotion in the No Pen (free mimicry) and Pen (mimicry blocking) conditions.*


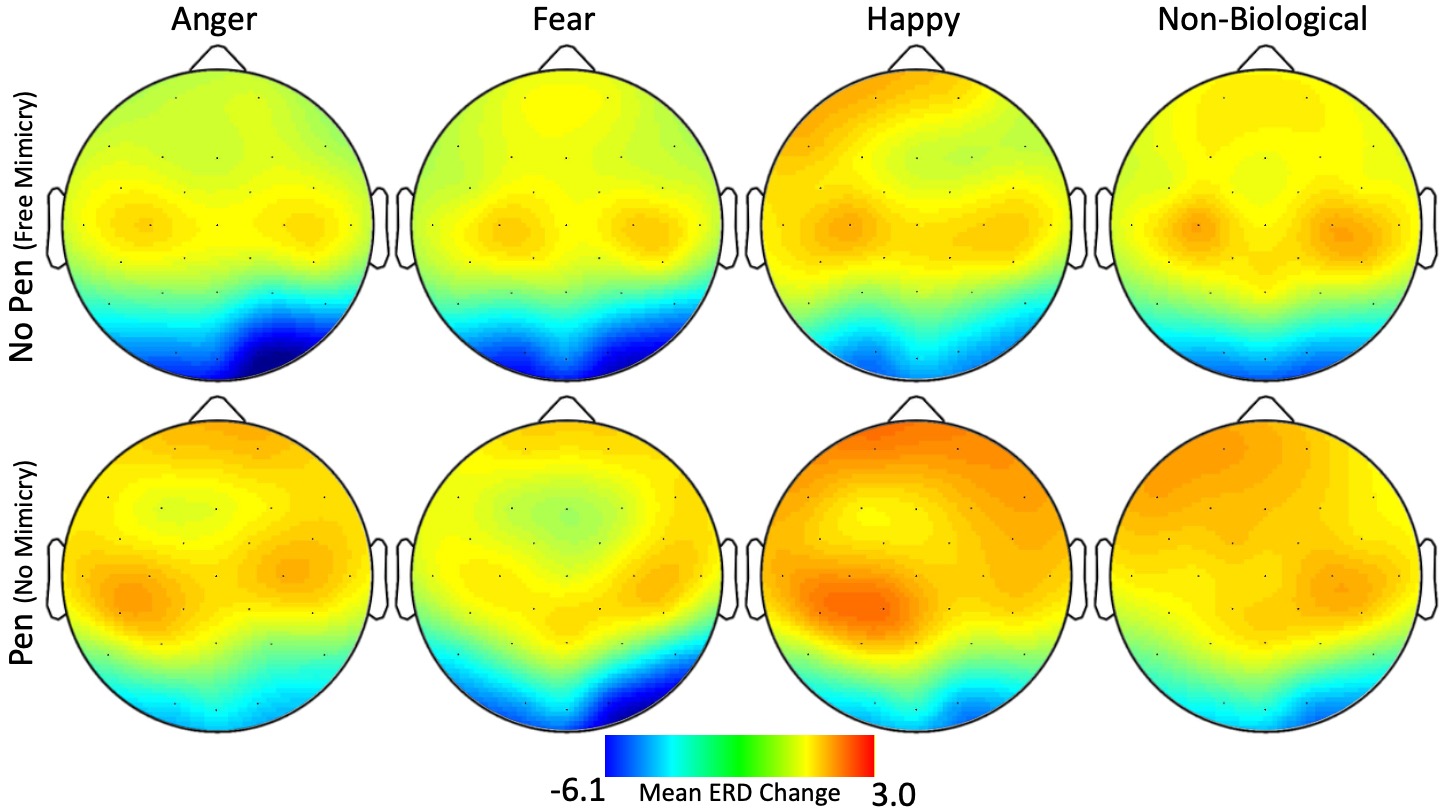

Supplement: Supplementary file 1 — (DOCX 1005 kb) [file 13415_2021_956_MOESM1_ESM.docx]
